# The horse Y chromosome as an informative marker for tracing sire lines

**DOI:** 10.1038/s41598-019-42640-w

**Published:** 2019-04-15

**Authors:** Sabine Felkel, Claus Vogl, Doris Rigler, Viktoria Dobretsberger, Bhanu P. Chowdhary, Ottmar Distl, Ruedi Fries, Vidhya Jagannathan, Jan E. Janečka, Tosso Leeb, Gabriella Lindgren, Molly McCue, Julia Metzger, Markus Neuditschko, Thomas Rattei, Terje Raudsepp, Stefan Rieder, Carl-Johan Rubin, Robert Schaefer, Christian Schlötterer, Georg Thaller, Jens Tetens, Brandon Velie, Gottfried Brem, Barbara Wallner

**Affiliations:** 1 Institute of Animal Breeding and Genetics, University of Veterinary Medicine Vienna, Vienna, 1210 Austria; 2 Vienna Graduate School of Population Genetics, Vienna, Austria; 3 United Arab Emirates University, 15551 Al Ain, UAE; 4 Institute for Animal Breeding and Genetics, University of Veterinary Medicine Hannover, Hannover, 30559 Germany; 5 Lehrstuhl fuer Tierzucht, Technische Universitaet Muenchen, Freising, 85354 Germany; 6 Institute of Genetics, Vetsuisse Faculty, University of Bern, Bern, 3001 Switzerland; 7 Department of Biological Sciences, Duquesne University, Pittsburgh, 15282 USA; 8 Department of Animal Breeding and Genetics, Swedish University of Agricultural Sciences, Uppsala, 75007 Sweden; 9 Department of Biosystems, KU Leuven, Leuven, 3001 Belgium; 10 Veterinary Population Medicine Department, University of Minnesota, St. Paul, MN 55108 USA; 11 Agroscope, Swiss National Stud Farm, Avenches, 1580 Switzerland; 12 Department of Microbiology and Ecosystem Science, Division of Computational Systems Biology, University of Vienna, Althanstrasse 14, 1090 Vienna, Austria; 13 Department of Veterinary Integrative Biosciences, College of Veterinary Medicine and Biomedical Sciences, Texas A&M University, College Station, TX 77843-4458 USA; 14 Department of Medical Biochemistry and Microbiology, Science for Life Laboratory, Uppsala University, Uppsala, 75123 Sweden; 15 Institut fuer Populationsgenetik, University of Veterinary Medicine Vienna, Vienna, 1210 Austria; 16 Institute of Animal Breeding and Husbandry, University of Kiel, Kiel, 24098 Germany; 17 Functional Breeding Group, Department of Animal Sciences, Georg-August-University Göttingen, Göttingen, 37077 Germany; 18 School of Life and Environmental Sciences, University of Sydney, Sydney, 2006 Australia

## Abstract

Analysis of the Y chromosome is the best-established way to reconstruct paternal family history in humans. Here, we applied fine-scaled Y-chromosomal haplotyping in horses with biallelic markers and demonstrate the potential of our approach to address the ancestry of sire lines. We *de novo* assembled a draft reference of the male-specific region of the Y chromosome from Illumina short reads and then screened 5.8 million basepairs for variants in 130 specimens from intensively selected and rural breeds and nine Przewalski’s horses. Among domestic horses we confirmed the predominance of a young’crown haplogroup’ in Central European and North American breeds. Within the crown, we distinguished 58 haplotypes based on 211 variants, forming three major haplogroups. In addition to two previously characterised haplogroups, one observed in Arabian/Coldblooded and the other in Turkoman/Thoroughbred horses, we uncovered a third haplogroup containing Iberian lines and a North African Barb Horse. In a genealogical showcase, we distinguished the patrilines of the three English Thoroughbred founder stallions and resolved a historic controversy over the parentage of the horse ‘Galopin’, born in 1872. We observed two nearly instantaneous radiations in the history of Central and Northern European Y-chromosomal lineages that both occurred after domestication 5,500 years ago.

## Introduction

The horse (*Equus caballus*) has accompanied humans ever since its domestication more than five millennia ago. While initially serving as a food source, the horse soon revolutionised agriculture, transportation and warfare. Vast empires were ruled from the back of the horse^1^. Today we count 58 million horses worldwide^2^ and distinguish about 700 breeds^3^. Extant horses are genetically clearly distinct from early domesticates^4^. Substantial turnovers in the horse genome coincide with the expansion and spread of human cultures starting from the Early Bronze Age over Scythian steppe riders and Roman times into the Middle Ages^5–7^. As such shifts were mainly achieved by breeding from limited numbers of stallions, they have led to a much-reduced male compared to female effective population size, explaining the difference in Y chromosome and mtDNA diversity^8^.

Strategic breeding in the past 300 years caused the most dramatic change in the genetic make-up of horses^9^. The formation of European and American breeds is marked by an enormous impact of imported stallions. According to written records, this process, intended as a refinement of local stocks, started in Europe with the popularity of Iberian sires from the 15^th^ to the 18^th^ century. It was followed by the so-called “Oriental wave” from the late 18^th^ to the late 19^th^ century. During this period “Original Arabian” stallions have been imported from Syria to Egypt to achieve speed and elegance. From the early 19^th^ century onwards, systematic upgrading of horse populations was mainly done through English Thoroughbred stallions. The establishment of most modern horse breeds attributes to this period^10^. Given the impact stallions had and still have to achieve breeding goals, a pedigree-independent, precise genetic analysis of sire lines is important.

The male-specific region of the mammalian Y chromosome (MSY) perfectly mirrors the patrilineage and MSY haplotype (HT) data have been widely used to infer the paternal ancestry of populations^11–16^. In humans, for example, the most used genetic markers to combine genetic data with family history are located on the MSY^17,18^. Tracing back the history of sire lines based on such genetic information, however, has been hampered by the low variability on the domestic horse MSY^19–21^. We recently demonstrated that MSY haplotyping can supplement pedigree information^22,23^. Based on a male genealogy inferred from an MSY reference spanning 1.46 million basepairs (Mbp) we showed that apart from an early branching Asian clade and a few other Asian and Northern European lineages, all Western domestic horses cluster in a recently established ‘crown haplogroup’^23,24^. We interpreted the predominance of the crown haplogroup in Western European and North American breeds as a consequence of the extreme preference of stallions of Oriental origin during the past few hundred years. Given the dominance of crown lineages in intensively bred horse populations, full understanding of the recent use of stallions requires a clear resolution of the crown haplotypes.

In this article, we generated a 6.46 Mbp sized reference of the horse MSY that goes far beyond the 1.46 Mbp non-repetitive MSY (nonrepMSY) used in previous studies. We further developed a probabilistic method to accurately define the regions on the repeat rich mammalian MSY^12^ that are suitable for unambiguous variant calling. By increasing the region screened for polymorphic markers and augmenting our previous dataset with more crown group breeds, we dissected the crown HT structure with a resolution only reached in human MSY studies so far. Our data permit new insights into the recent history of horse breeding.

## Materials and Methods

A detailed description of methods including program parameters and program versions used is available in the Supplementary Information.

### Ethics statement

The study was discussed and approved by the institutional ethics and welfare committee of the University of Veterinary Medicine Vienna in concordance with GSP guidelines and national legislation (ETK-10/05/2016). The research was performed in accordance with relevant guidelines reported in the above-mentioned document. All samples are coded and an informed consent was obtained from horse owners.

### Samples and raw data processing

#### Next generation sequencing (NGS) data

Whole-genome Illumina data from 130 male domestic horses, nine Przewalski’s horses, one donkey and five female horses used in the study were either obtained form previously published studies^23–35^ and downloaded from publicly available sources or sequenced as part of this study. Details on samples are given in Supplementary Table S1 and Supplementary Fig. S1. Removal of adapter sequences and a quality-based trimming was performed for all samples using ReadTools^36^.

#### DNA samples used for genotyping

Hair root samples from male horses were derived from breeding associations and private horse owners. Genomic DNA was isolated using DNeasy Blood and Tissue Kit (Qiagen®).

#### Y chromosome de novo assembly

Prior to the MSY *de novo* assembly we reduced the complexity of the input data. Therefore, we used a bioinformatics-based approach to enrich paired end Illumina short reads of three whole-genome sequenced Lipizzan stallions (sample IDs Lip111, Lip113 and Lip169 in Supplementary Table S1) for Y-specific sequences. We used published Y-chromosomal sequences – the 1.6 Mbp nonrepMSY contigs^23^, six BAC-clones^22^, human and mouse Y-chromosomal sequences (NCBI GenBank), and equine Y-chromosomal and XY-homologous GenBank entries^27,37^ as baits to identify putatively Y-specific reads. A detailed description of the Y read enrichment step is given in the Supplementary Information and bait details are listed in Supplementary Table S2. We mapped each Lipizzan male with bwa aln^38^ to a file with multiple FASTA-formatted sequences containing all the bait sequences. We then extracted mapped read-pairs using samtools^39^ and used them as input for the generation of a *de novo* assembly with SPAdes^40^. Contigs shorter than 200 bp were discarded. We used REAPR^41^ to correct for assembly errors. Details are shown in the Supplementary Information.

### Classification of scY, mcY and nonMSY windows

Using a probabilistic model, we classified the assembly into single-copy (scY), multi-copy (mcY) Y and not MSY (nonMSY) regions (X-chromosomal, autosomal, pseudoautosomal or XY-homologous regions) based on coverage differences between females and males in 50 bp windows.

#### Preparing input data for the classification

Whole-genome Illumina data from five females and ten males (samples indicated in Supplementary Table S1) were mapped to the REAPR-corrected assembly and the equine X chromosome with bwa aln^38^. Per-site mean coverage of each 50 bp window and horse was determined using Unix commands, Python^42^ and bedtools^43^. As sequencing-depth differs among individuals (see Supplementary Table S1, Supplementary Fig. S1), we normalised each window’s mean coverage: an individual’s diploid coverage was inferred as its mean coverage of windows in the pseudoautosomal region (PAR) of the X chromosome. For each horse, the mode of the distribution of the mean coverages of the PAR windows was calculated in R^44^ and used to normalise the mean coverage per window in the Y chromosome assembly, such that a relative coverage of one corresponds to a diploid state. Details are given in the Supplementary Information.

During visual inspection of alignments, we noted that reads from females spuriously map to single-copy MSY regions at a background level. Each female’s background coverage was estimated as its mean normalised mapping coverage in confirmed single-copy MSY windows. These single-copy MSY windows were regions to which the single-copy MSY contigs from the previously published nonrepMSY reference (GenBank accession MPVR00000000)^23^ mapped using bwa mem^38^ with the default settings.

#### Probabilistic model

The normalised coverages in 50 bp windows were modelled as Poisson distributed. Using R^44^, each Y assembly window was assigned to one of two classes: i) MSY or ii) nonMSY. The observed mean coverages per window *k* and horse (*i* for males and *j* for females; provided to get the normalised mapping coverages *y*), each horse’s mean mapping coverage of the PAR *c* and the female background coverages *b*, all obtained as described above, are provided as input for the R script. Assuming equal prior probabilities, the probability of assignment of a window to class i) or ii) can be calculated from this ratio (the R script and derivation of the formula are given in the Supplementary Information):

$$\frac{Pr({y}_{i,k},{y}_{j,k}|{\hat{v}}_{k},b,c)}{Pr({y}_{i,k},{y}_{j,k}|{\hat{\mu }}_{k},c)}=\frac{{\prod }_{i=1}^{I}{({\hat{v}}_{k}{c}_{i}/2)}^{{y}_{i,k}}{e}^{-{\hat{v}}_{k}{c}_{i}/2}{\prod }_{j=1}^{J}{b}_{k}^{{y}_{j,k}}{e}^{-{b}_{k}}}{{\prod }_{i=1}^{I}{({\hat{\mu }}_{k}{c}_{i})}^{{y}_{i,k}}{e}^{-{\hat{\mu }}_{k}{c}_{i}}{\prod }_{j=1}^{J}{({\hat{\mu }}_{k}{c}_{j})}^{{y}_{j,k}}{e}^{-{\hat{\mu }}_{k}{c}_{j}}}$$

**Figure 1:**
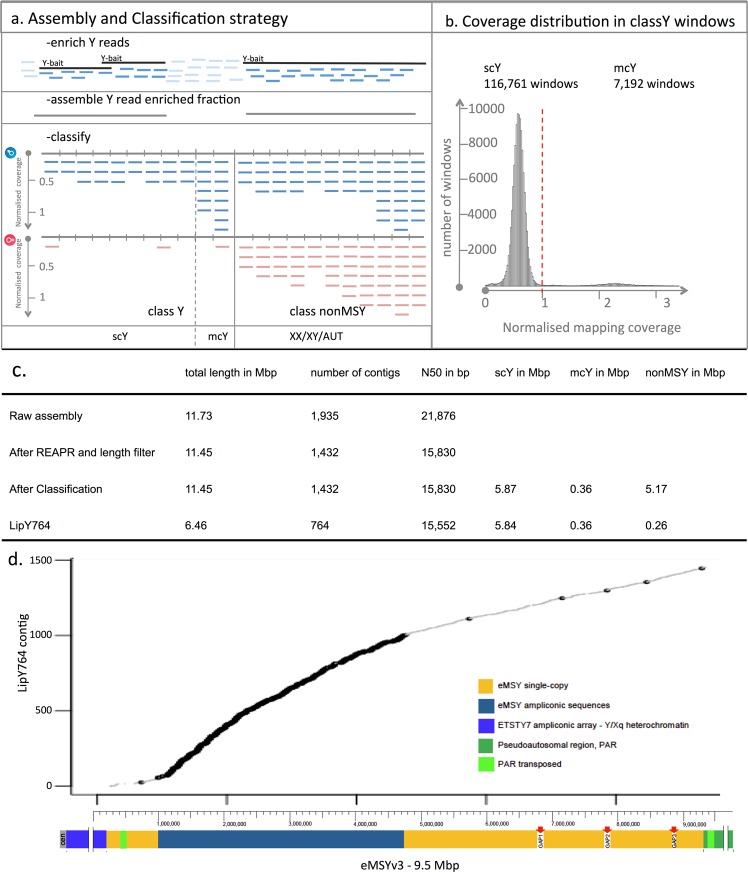
LipY764 Assembly. (**a**) Assembly step: whole genome NGS reads from males (blue) are mapped to Y-specific bait sequences (black). Mapped reads (dark blue) are then extracted and assembled (grey). Classification step: the assembly is shown in grey with hatchmarks representing 50 bp windows. Male (blue) and female (red) reads are mapped to the assembly and mapping coverages normalised to autosomal coverages per window were estimated. The probability of Y- or nonMSY-specificity per window is obtained by comparing normalised coverages in males and females. class nonMSY: XX/XY/AUT ≈ 1 in males and ≈ 1 in females; class Y: scY ≤ 1 in males and ≈ 0 in females, mcY > 1 in males and ≈ 0 in females. (**b**) Frequency distribution of normalised mapping coverage in classY windows. The cut-off scY to seperate mcY is set to 1 (red dashed line). (**c**) Resulting statistics for the assembly and classification approach. (**d**) Position of LipY764 contigs on eMSYv3^46^ (data in Supplementary Table S5). Contigs having a single unique position on eMSYv3 are shown in grey, contigs with multiple hits in black/bold.

According to the observed distribution of windows assigned to class i), a relative coverage cut-off value less than one was selected to distinguish scY from mcY windows in the MSY fraction (see Fig. 1).

### LipY764 predefinition

Contigs with a Y-specific content less than 45% were discarded from the assembly (see Supplementary Fig. S2). After filtering for contig length ≥ 300, the remaining classified 764 Y-specific reference contigs were further characterised (see below) and released (LipY764, PRJNA428358). The scY and mcY coordinates are provided in Supplementary Table S3 and other contig details are given in Supplementary Table S5. Summary statistics of the final assembly were computed with Python^42^.

### LipY764 comparisons and gene content

BLAST^45^ was used to align the nonrepMSY reference^23^ to the assembled contigs. BLAST^45^ was also used to compare the LipY764 contigs with eMSYv3^46^ by setting the thresholds for a match to nident > 299 and pident > 95%.

### Horse Y phylogeny

A horse Y phylogeny was generated using variants ascertained from whole-genome NGS data of 139 males (Supplementary Table S1).

### Variant ascertainment and validation

The FastQC-checked, adapter free and trimmed data were mapped to the assembly using bwa aln^38^. PCR duplicates and low quality mappings were filtered with samtools^39^. Variant calling was performed using HaplotypeCaller and CombineGVCFs from GenomeAnalysisTK^47^ (details in the Supplementary Information). As CombineGVCFs does not detect the full diversity when using a sample set with uneven haplotype distribution (for example many modern domestic and few Przewalski’s horses) we ran CombineGVCFs for several sample compositions (for example only the Przewalski’s horses, the deep-branching Asian horses or the crown group) to ensure valid calling also for HTs underrepresented in our dataset. Several filtering steps were performed on the outputs using Python^42^ to predict a final list of high quality biallelic variants. First, only variants found in scY windows were considered and then phased variants, variants with multiple alternatives and reference errors were excluded. In the third step a read depth of at least three in one individual and a genotype quality ≥ nine were set as limit to keep the variant in the list. In the last step variants with heterozygous or no-calls in more than 10% of the samples were excluded. A final CombineGVCFs run with all samples was performed to implement the identified variants for all samples. Still ambiguous or missing variants for low-coverage samples were subsequently corrected according to their phylogenetic clustering. All positive single nucleotide variants (SNVs) and insertions or deletions (indels) detected in the Dom-West horses were checked visually in IGV^48^, by comparing the site in multiple samples in parallel. 166 LipY764 variants (indicated in Supplementary Table S8) are already lab validated using LGC KASP technology, as described previously^23^.

### Phylogeny

Allelic states of 2,193 variants (2,035 described herein the first time; Supplementary Table S8) were catenated to construct MSY haplotypes. The ancestral variant state was inferred from the donkey or the Przewalski’s horse for variants polymorphic only in domestic horses. A maximum parsimony (MP) tree rooted with the donkey was generated with PAUP^49^ and bootstrapping with 1,000 replicates was performed. Further, a maximum likelihood (ML) tree was reconstructed with RAxML^50^ (-m gtrgamma) with 1,000 bootstrap replicates. Trees were finalised using FigTree^51^. The topology and frequencies of the MSY haplotypes were additionally visualised with Network^52^.

### eMSYv3 variant coordinates

With BLAST^45^ and Python^42^ the variant coordinates were lifted over to eMSYv3^46^ based on 100% identity of the flanking sequences (Supplementary Table S8). For variants on contigs with a single BLAST hit on eMSYv3^46^ (Supplementary Table S5), whose positions were not detected with Python^42^, the positions on eMSYv3^46^ were inferred manually by allowing mismatches in the flanking region using CLC Genomics Workbench^53^.

### Microsatellite analysis

109 individuals were genotyped for the tetranucleotide microsatellite fBVB (Supplementary Table S9, for details see Supplementary Information). Genomic DNA was isolated from hair roots using nexttec®. One primer was labelled with FAM fluorescent dye to allow analysis of fragments (fwd_FAM: ACAACCTAAGTGTCTGTGAATGA; rev: CCCAATAATATTCCACTGCGTGT, expected amplicon length 204 bp) on an ABI 3130xl Genetic Analyzer. PCR was performed in a 20 µl volume containing 0,4 µM of each primer. The DNA was initially denatured at 95 °C for 5 min, followed by 35 cycles of 30 s at 95 °C, 40 s at 58 °C annealing temperature and 40 s at 72 °C, and a final extension of 30 min at 72 °C. Alleles were sized relative to the internal size standard using GeneMarker®.

### Pedigree reconstruction and generation time intervals

We inferred the paternal genealogy for 333 males using pedigree information provided by breeding associations or matched information from several web-based databases (listed in Supplementary Information). Horses with inconsistent genealogical records across different databases were discarded. The reconstruction revealed 1,722 father-son pairs; the years of birth of sons range from 1680 to 2000. MSY haplogroups (HGs) of these 333 individuals were inferred by either whole genome sequencing or KASP genotyping (see Supplementary Information).

### *De novo* mutation rate and branch length estimates

We generated a maximum parsimony tree using only samples with a mean single-copy Y coverage ≥ six, considered only SNVs (Supplementary Table S8) and counted the number of mutations on branches. We tested pairs of branches of MSY haplotypes descending from the same split for equality of their numbers of mutations assuming a Poisson distribution with the same expectation using a chi-square test of equal frequencies and determined p-values by 10,000 simulations in R^44^. Correlation between branch length and mean generation interval was tested with a chi-square test for given probabilities with simulated p-value (based on 10,000 replicates, see Supplementary Information). We inferred a mutation rate with Darley Arabian, born in 1700 as calibration point. From pedigrees of 592 father-son pairs tracing back to Darley Arabian, on average 28.3 generations were counted in 320 years, resulting in a mean generation interval of 11.36 years.

We estimated a *de novo* mutation rate from our data by dividing the mean observed *de novo* mutations (2.75) per 28.3 generations by the length of the single-copy MSY sequence (5.83 Mb).

### Molecular dating

We dated the most important nodes with BEAST^54^ assuming a mutation rate of 1.69*10^−9^ mutations/site/year, which is based on the rate/site/generation calculated from our data (see results section) and a ten years generation interval. We generated a confidence interval allowing for plus/minus two years generation interval resulting in 2.11*10^−9^ mutations/site/year for eight and 1.41*10^−9^ mutations/site/year for twelve years generation time. The substitution model to best fit the data was chosen according to datamonkey^55^ (HKY and gamma site heterogeneity). We used a constant-sized coalescent tree prior and a strict clock. A prior with a normal distribution based on the 95% CI of the substitution rate was applied. Only the variant sites were used and the number and composition of invariant sites was defined in the BEAST xml file. Markov chain Monte Carlo (MCMC) samples were based on 20,000,000 generations, logging every 1,000 steps. Two runs were combined using BEAST’s^54^ LogCombiner and TreeAnnotator with the first 10% discarded as burn-in. The final tree was visualised using FigTree^51^.

## Results and Discussion

### Generation of a Y chromosome draft assembly

Y-chromosomal regions are often underrepresented in whole-genome shotgun assemblies^56^. Tomaszkiewicz *et al*.^57^ efficiently assembled gorilla Y-chromosomal regions by flow-cell sorting the Y chromosome prior to Illumina sequencing. We mimicked the flow-cell enrichment computationally and generated a Y-enriched short read dataset by mapping whole-genome NGS data from three Lipizzan males (Supplementary Table S1) to a collection of publicly available Y-specific sequences (details in Supplementary Table S2). In total, 2,549,458 read-pairs mapped to the captured sequences and these reads were used for generating the raw assembly. The raw assembly had 1,935 contigs and a total size of 11,727,306 bp (with contig lengths between 56 and 112,142 bp and an N50 of 21,876 bp).

Next, we developed and applied a probabilistic method to classify the assembly into i) single-copy (scY) and multi-copy (mcY) MSY regions and ii) not Y-specific (nonMSY) regions based on different mapping coverages in males and females (see Material and Methods). An overview of the assembly and classification strategy including assembly statistics is shown in Fig. 1. After the classification and filtering steps, 764 non-overlapping contigs with a total size of 6.46 Mbp remained (LipY764; SAMN08288327). We classified 5.84 Mbp of LipY764 as scY, 0.36 Mbp as mcY and 0.26 Mbp as nonMSY (region details in Supplementary Table S3). The assignment of windows to class i) or ii) is strongly influenced by the mapping settings and complexity of the reference. Most scY windows are expected to be located in X-degenerated regions in old evolutionary strata^58^ with high sequence divergence to the X gametolog.

The low repetitive content in our 6.46 Mbp MSY assembly can be explained by difficulties of the assembler to bridge highly repetitive regions based on short read information only^59^. A similar method, the ‘chromosome quotient method’, to classify single-copy regions on the hemizygous sex chromosome has been proposed earlier^60,61^. As our probabilistic model inferred posterior probabilities of assignment to scY, mcY, and nonMSY in 50 bp windows, we achieved a refined classification with little loss of information caused by false negatives. At first glance, LipY764 corresponds to 54% of the 12 Mbp euchromatic region proposed for the horse MSY^46^. Tomaszkiewicz *et al*.^57^ doubled the size of their 13.3 Mbp short read Illumina assembly of the gorilla Y chromosome with mate-pair and PacBio data. However, as the ampliconic regions added by such efforts would not significantly increase the power for variant ascertainment and haplotype analysis, we did not attempt to further extend the assembly.

The finding that 2,765 of the 2,794 nonrepMSY contigs (GenBank accession MPVR00000000) used in previous studies^4,23^ were embedded in LipY764 (Supplementary Table S4) confirms that LipY764 is an improvement of the nonrepMSY assembly. Very recently, Janečka *et al*.^46^ published the first comprehensive assembly of the horse MSY with a total length of 9.5 Mbp, by sequencing 192 BACs from a BAC tiling path map (eMSYv3, GenBank accession MH341179).

This offered us the unique opportunity to assess the quality and complexity of LipY764 in a direct comparison. We found perfect eMSYv3 homologies for 585 LipY764 contigs (4.9 Mbp in total; Supplementary Table S5). When we allowed for multiple matches on eMSYv3, LipY764 covered even 7.05 Mbp (74%) of eMSYv3 (Fig. 1d). Most LipY764 contigs with multiple matches were already classified as mcY over most of their length (Supplementary Table S5). The 1,122 gaps were evenly distributed along eMSYv3 (Supplementary Table S6) and gap lengths in the X-degenerate and the multicopy region of eMSYv3 ranged from 1 to 150,228 bp (median = 503 bp, 1^st^ qu. = 163 bp, 3^rd^ qu. = 1,408 bp).

Interestingly, 179 contigs (1.48 Mbp) were not found on eMSYv3 (which is based on a Thoroughbred), but were unique to LipY764 (Supplementary Table S5). These contigs were evenly covered by NGS reads in all males analysed, including Thoroughbreds. Hence, they are not large insertions in the Lipizzans used for the LipY764 assembly, but rather represent regions not yet assembled and potentially informative for closing the remaining gaps in eMSYv3.

The 9.5 Mbp eMSYv3 harbours about 4 Mbp that are not accessible for variant detection using short read data^46^, such as the ampliconic region, the ETSY7 array, parts of the PAR and the PAR transposed region. For the ascertainment of variants to trace male lineages, LipY764 is advantageous because it contains 1.48 Mbp unique sequences and has therefore a larger total amount of scY regions. In contrast, only 853 kbp of the X-degenerate region of eMSYv3 are not covered by any LipY764 contig (Fig. 1, Supplementary Table S6). Altogether, the high concordance between the two references proves the reliability and the achievements we made with the more cost-effective and rapid approach we used to generate LipY764.

Of the 174 transcripts annotated on eMSYv3, 85 (48%) were covered to at least 95% on LipY764. Of the rest, 35 (20%) transcripts were partially and 54 (31%) not at all represented on LipY764 (Supplementary Table S7). Among the missing genes were those identified as recently transposed from the PAR to the MSY^46^ (Fig. 1, light green) and many other MSY genes with high sequence similarity to X gametologs or autosomal paralogs (Supplementary Table S7). With our algorithm, such regions would be classified as X-chromosomal or autosomal and removed during the classification step from downstream analyses.

### Fine-scaled Y-chromosomal haplotype structure in domestic horse breeds

**Figure 2:**
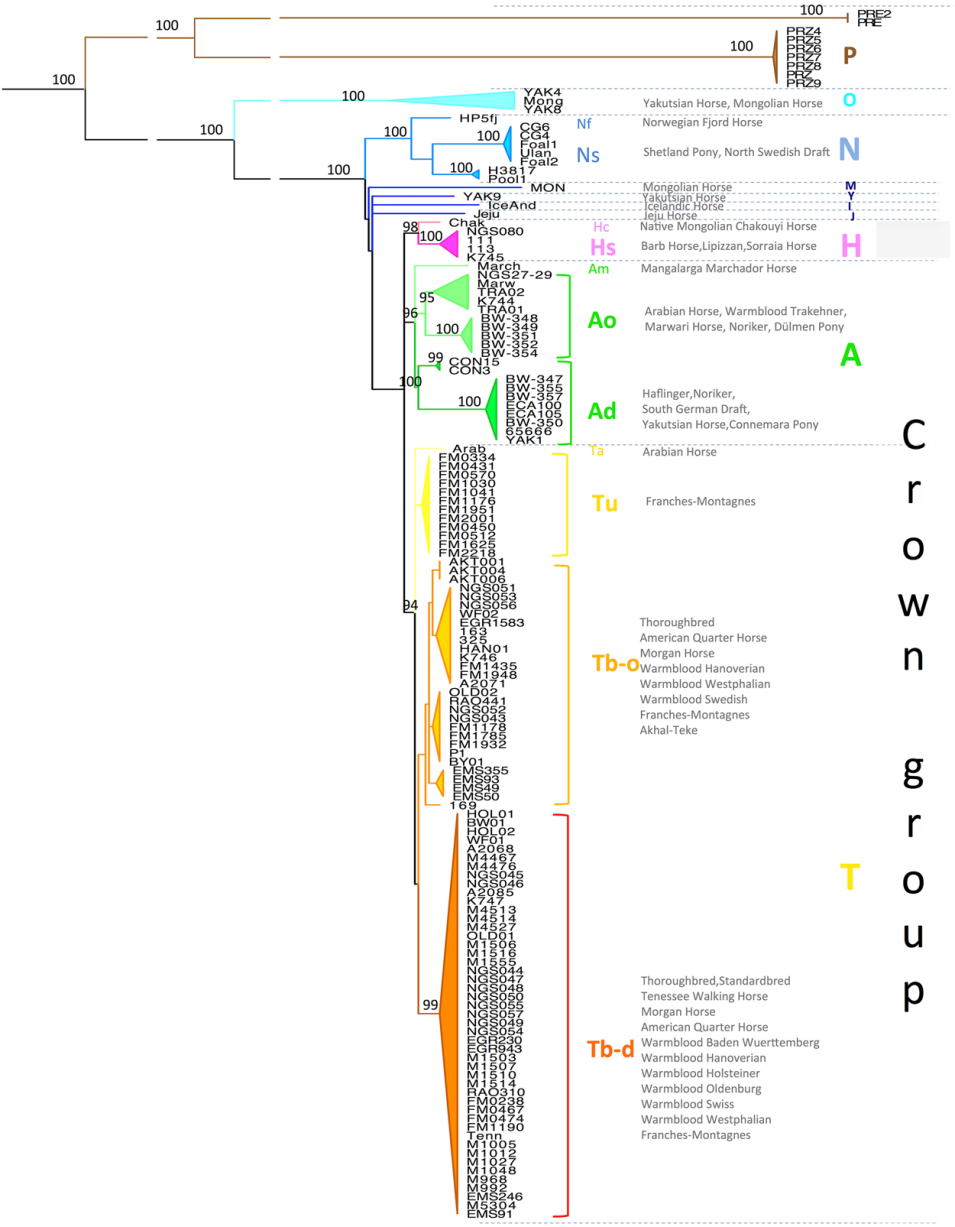
Horse MSY tree. A maximum parsimony tree showing the horse MSY phylogeny based on 2,192 scY variants detected in 139 males. The tree is rooted with the donkey and bootstrap values of 90% or higher are shown. The Przewalski’s horses are shown in brown. Blueish clades correspond to early splitting Asian samples (O), Northern European breeds (N and I) and other autochthonous Asian samples (M, Y and J). The three clearly separated crown group clades are represented in pink (H), green (A) and orange (T) shades. Assigned haplogroups are shown on the right. A detailed haplotype network with variants is shown in Supplementary Fig. S4, variant details are given in Supplementary Table S8.

We ascertained SNVs and small indels only in unambiguous scY windows of LipY764 predicted by the probabilistic model. By doing so we stringently excluded multi-copy and X-chromosomal/autosomal homologous regions, where unambiguously mapped reads could lead to inaccurate variant prediction (Supplementary Fig. S3). In total, we screened 139 whole-genome NGS sequenced males (see Methods) and ascertained 2,187 variants (2,027 SNVs, 159 indels and one microsatellite; see Supplementary Table S8), of which 152 have been already described previously^22–24^. Our dataset covers most Central European breeds. In addition to the 104 horses from Felkel *et al*.^24^ we analysed eleven Thoroughbreds, 16 horses from Western European breeds and eight Przewalski’s horses (sample details in Supplementary Table S1, Supplementary Fig. S1). Based on the 2,187 variants detected on LipY764 and implementing allelic states from six additional variants described previously^24^ (of which one is invariant in our dataset), we distinguished 76 haplotypes. Within domestic horses, we observed 71 haplotypes determined by 740 variants (735 on LipY764 plus the five additional variants); the remaining 1,452 variants separated the Przewalski’s horses from domestic horses. On the horse MSY, coalescence times are so recent that no double mutations at any site were detected and MP can be used to infer the genealogy (Fig. 2; corresponding network in Supplementary Fig. S4).

The phylogenetic trees (Fig. 2, Supplementary Fig. S5) correspond to previous results^23,24^ based on the shorter nonrepMSY: the Przewalski’s haplogroups (HG) Pz-a and Pz-b are clearly separated from the domestic horses. Within the domestic horses a few Asian samples branch early (HG O) and are connected to the crown via few Northern European (HGs N and I) and other Asian lines (HGs J, M and Y).

As expected, most modern horse breeds (115 horses or 83% of all samples) clustered in the previously described crown haplogroup (Fig. 2). This held true even for the Dülmen Pony, an ancient autochthonous Central European horse^10^. Within the crown, we distinguished 58 HTs defined by 211 variants. As the number of crown HTs almost doubled, we switched to human guidelines for MSY haplotype nomenclature^62^, but kept the first four letters consistent with the previous studies^23,24^. We now resolved three haplogroups forming a trichotomy at the base of the crown group. Apart from the previously described clades A (green in Fig. 2) and T (yellow in Fig. 2)^22,24^, we now resolved a new HG H (pink in Fig. 2) characterised by four variants (fRO, fRO, fYR, fBZU; Supplementary Fig. S4). All HG H samples except the Barb horse (subgroup Hs) were analysed previously^24^ and formed independent groups (S - Sorraia horse, L - Lipizzan horses and C - Chinese Chakouyi horse). The history of breeds representing subgroup Hs so far (Barb horse, Spanish breeds, Sorraia horse, Baroque breeds) suggests that this HG entered Europe from the West via Spain by the introduction of North African horses^1,10,63^.

### The microcosmos of the Tb-clade and English Thoroughbred sire lines

**Figure 3:**
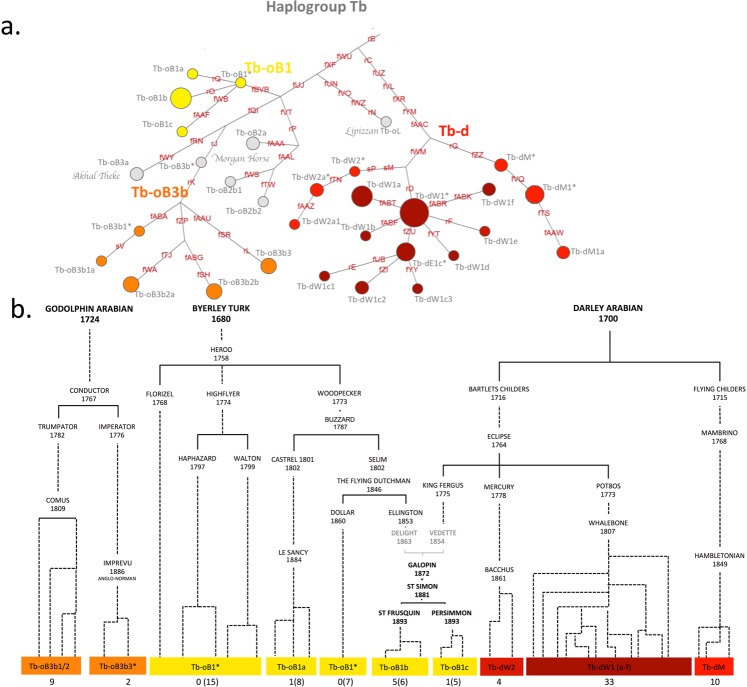
Detailed view on haplogroup Tb. (**a**) Haplotype network of group Tb. Circle sizes correspond to the number of samples. Nomenclature of HTs is based on Wallner *et al*.^23^ and subbranches according to human guidelines^62^. Determining variants are given on branches (details in Supplementary Table S8). HTs derived from Darley Arabian are shown in red, Godolphin Arabian in orange and Byerley Turk in yellow. (**b**) Pedigree reconstruction of English Thoroughbred descendants and the respective HTs. Dotted lines connect relatives where at least one ancestor is omitted. For each HT the number of samples in the NGS dataset is given and the number of genotyped individuals, if available (Table S9), shown in parentheses.

More than half of the domestic horses in our dataset (76 of 130) carried a haplotype of HG Tb. These included English Thoroughbreds, Standardbreds, many Thoroughbred-influenced breeds (Warmbloods, American Quarter horses, Franches-Montagnes), a Lipizzan stallion, and the Akhal-Tekes. Previously, we identified HG Tb as a signature of the Turkoman horse, an ancient horse population from the steppes of central Asia^23^. During the past 300 years HG Tb was extensively spread by the English Thoroughbred. The Thoroughbred sire lines trace back to three founder stallions that were imported to England at the end of the 17^th^ century^10,64^. Here, we fully resolved the heritage of the Thoroughbred sire lines with MSY haplotyping. The haplotype structure of 65 males was highly consistent with their paternal genealogy inferred from the pedigree (Fig. 3). We now clearly discriminate discrete sublines of Darley Arabian, born in 1700 (Tb-d) and Godolphin Arabian, born in 1724 (formerly Tb-g, now Tb-oB3b). The third founder, Byerley Turk, born in 1680, was newly characterised by an allelic variation of the tetranucleotide microsatellite fBVB (GATA_14_/GATA_15_; Supplementary Table S8) that defines the Tb-oB1 clade. According to pedigree information only few of the tested males trace back paternally to Byerley Turk. We thus screened a representative dataset of 109 purebred males by genotyping fBVB and detected allelic variation of fBVB only in Tb clade horses (Supplementary Table S9). All 30 patrilineal descendants that coalesce in Herod, born in 1758, whose ancestry in turn traces back to Byerley Turk, carried the Tb-oB1 specific allele 208 (Supplementary Table S9). We also confirmed Tb-oB1 in eleven horses tracing back to St. Simon, born in 1881. According to stud records, St. Simon was the son of Galopin, born in 1872, who was sired by Vedette, born in 1854. The line should trace back to Eclipse, born in 1764 (Fig. 3) according to stud books. All descendants of St. Simon carry Tb-oB1, undoubtedly the HT of Herod (Fig. 3), and not that of Eclipse (Tb-dW*). Thus, an incorrect paternity assignment must have occurred in this lineage. In a discussion recorded in the early to mid 19^th^ century, one party claimed that instead of Vedette a moderate performer named Delight, born in 1863, a Byerley Turk descendant (Fig. 3b), fathered Galopin^65–67^; our molecular data support this view.

In addition to the patrilines of the English Thoroughbred, we distinguished Tb-oB3a, Tb-oB2a, and Tb-oL represented by the Akhal-Tekes, the Morgan horses and a Lipizzan horse in the Tb clade (Fig. 3a). The private clustering of these lines suggests an origin from a similar source population but independent from the Thoroughbreds.

### Potential and perspectives for individual MSY haplotyping in horses

In humans analysis of the MSY is well-established in forensics^68^. Due to the Y chromosome being a single locus and mirroring the genealogy of only the male sex, its analysis allows only limited conclusions about the rest of the genome. However, as the male side plays such an important role in horse breeding, MSY genealogies reveal not only paternal ancestry of horses but also breeding history in general. Our fine-scaled resolution of the individual Thoroughbred lines underlines that horse MSY haplotyping is a practically valuable and accurate method to assess male ancestry. By further augmenting the haplotype network with sire line representatives from other classical refiner breeds (e.g., the imported Arabians and the Iberian/North African horses), our knowledge of the development and history of present-day horse breeds can be further improved. Horses with doubtful ancestry can now be assigned to sire lines and recent male-driven introgressions can be documented in detail^69^.

Currently we have no indication of fitness effects of the variants reported here. Four of the 24 variants in transcribed regions (Supplementary Table S8) were in UTRs and thus not coding. Of the remaining 20, only five were polymorphic in domestic horses, the other 15 separate Przewalski’s and domestic horses. Due to the limited number of candidates and the lack of phenotypic data, we refrained from further functional investigation. So far, the major phenotypic effects (mainly reduced fertility) described for the Y chromosome are due to structural and copy number variations^12,70–72^, which we did not examine in the context of this work.

### Dating historic horse radiations

The high mtDNA diversity observed in modern domestic horses reflects the preservation of diverse wild ancestral maternal lineages^73,74^. In contrast, most ancient MSY variation^5,8^ was lost and the low MSY variation in modern domestic horses mirrors a very recent origin of extant sire lines. Knowledge of the exact timing and location of the emergence of the predominant MSY lineages would allow identification of the corresponding human cultures and improve our understanding of the human-horse history.

**Figure 4:**
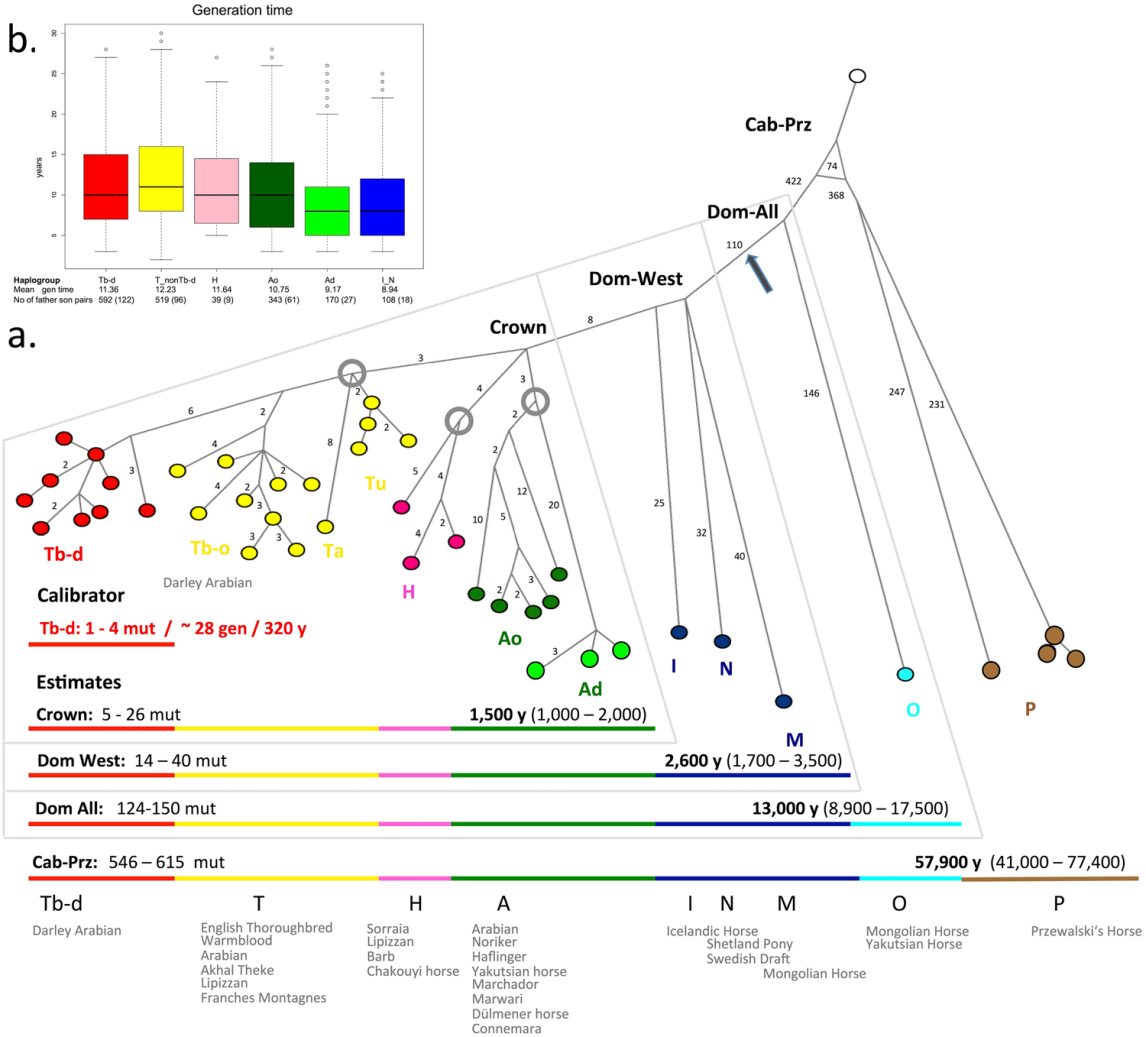
Divergence time estimates. (**a**) Maximum parsimony tree rooted with the donkey. Coloured circles represent contemporary haplogroups; grey rings indicate basal HTs of the crowns’ sublines. The number of mutations on a branch is given on its left, unless it is one. In the lower panel the full range of mutations observed after respective coalescence points (mut) and years back to the MRCA (y) under the assumption of a mutation rate of 1.69 × 10^−8^ site/generation and assuming a generation interval from eight to twelve years (**b**) is shown. 95% highest posterior density intervals are given in brackets. The position of variant fBOI (indicative for Y-HT1 in Wutke *et al*.^77^) is marked by an arrow. Details on variants are given in Supplementary Table S8. (**b**) Mean generation intervals calculated from deep pedigrees from males genotyped for the respective haplogroup. The number of father-son pairs is given with genotyped individuals in parentheses (data in Supplementary Table S10).

We detected two early splits in the MSY phylogeny, one separating the Przewalski’s from modern domestic horses (Cab-Prz) and the other between Western and Asian haplotypes (Dom-All). Additionally, we observed two recent consecutive radiations: ‘Dom-West’, encompassing all non-Asian domestic horses with the deepest branch leading to the Shetland Pony and the North Swedish Draft Horses (N), followed by the more recent crown radiation (Fig. 4a). We postulate that the dispersal of the crown started from a population already harbouring the basal HTs of the crown’s sublines (grey rings in Fig. 4a). The ancestors of the classical refiner populations - North African and Iberian, Arabian, and Turkoman horses - likely originate from this ancestral pool. Additionally, a Chinese lineage falls into this group.

The unbiased variant ascertainment in our approach would allow for exact dating of coalescent times under the assumption of a molecular clock: with a constant mutation rate and identical generation intervals, branches descending from one split are expected to accumulate new mutations at the same constant rate.

For dating, we omitted low-coverage samples, did not consider indels (see Methods) and inferred the genealogy from 42 HTs based on 1,856 variants assuming parsimony (Fig. 4a). To test for the expected equality in branch lengths, we assumed a Poisson distribution (see Methods). We found significant deviations from an equal distribution of mutations on individual branches within recently emerged haplogroups, e.g., between branches in Dom-West (14 to 40, X^2^ = 34.532, p-value < 0.01) and branches in the crown (five to 26 mutations; X^2^ = 34.55, p-value < 0.01). No such deviation was observed when contrasting number of mutations accumulating from older branching points (dom_all, dom_prz). Most obvious was the contrast between the exceptionally long Ad-h branch (Noriker Coldblood horse) and the shorter Tb branches (Thoroughbred).

Overestimation of branch lengths could have been induced by several mutations that occurred simultaneously due to X-Y gene conversion^22,75^ but were by chance not marked as phased variants by GATK. These variants would have been retained in our final variant list and by mistake assumed as independent. Additionally, it could be that the X chromosomes of our five females are too dissimilar from potential gene conversion regions such that our method could not identify them as nonMSY regions. We ruled out X-Y gene conversion as causative for the long branches, since we did not observe a signature of closely clustering variants occurring on the same contig (Supplementary Fig. S6, Supplementary Table S8).

As stated above, the excess of variation could also be due to varying generation times. Mean generation intervals in father-son pairs inferred from deep rooting pedigrees, genotyped for respective HGs (Supplementary Table S10), followed the trend observed in branch lengths (Fig. 4b). Coldblooded males, often carrying Ad, tended to have shorter mean generation intervals (8.94 years) than horses carrying Tb (mean 11.3 to 12.23 years; Fig. 4b) in the past 300 years. We tested the two most extreme branches, namely Ad-h and T carriers; differences in generation intervals were insufficient to account for the different numbers of mutations (X^2^ = 8.6541, p-value < 0.01).

The violation of the assumption of a constant mutation rate per unit time compromised dating. Nevertheless, we estimated the time to most recent common ancestors (MRCA; Fig. 4b). We calibrated the mutation rate using mutations after Darley Arabian (Tb-d). The resulting mutation rate (1.69 × 10^−8^ mutations/site/generation or one *de novo* mutation every 10.2 generations in the MSY region under investigation) was lower than the previous estimate (2.91 × 10^−8^ mutations/site/generation) based on four *de novo* mutations in paternal families^23^, but agreed much better with genome-wide estimates in humans^76^. Despite the wide range of our estimates (Fig. 4b, Supplementary Fig. S7), we confirmed that all patrilineal Western horse lineages (Dom-West) arose after domestication (MRCA 1,700–3,500 years before present) with the crown embedded therein (MRCA 1,000–2,000 years before present). The analysis of dated archaeological samples will make it possible to more precisely determine the cultural and biogeographic origin of Dom-West and the crown, and to more thoroughly resolve paternal ancestry of horses. In a recent study based on a collection of ancient samples capturing nearly the entire history of horse domestication^77^ it was revealed that a single Y-HT, first observed in a 4,200 year old sample, invaded and spread in the population until it reached fixation. The SNV fBOI, defining the ‘fixation Y-HT1’ in Wutke *et al*.^77^, is one of the 110 variants separating Dom-West from the Przewalski’s horse and the Asian haplogroup (indicated by an arrow in Fig. 4b, details in Supplementary Table S8). The signal of Y-HT1 in Wutke *et al*.^77^ therefore comprises all Dom-West HTs and its emergence is consistent with our dating estimate.

## Conclusion

Until recently, the only means to trace sire lines were often incomplete or even erroneous pedigree data. Here, we resolved the MSY sequence variation of horses at a resolution comparable to that in humans. We have enabled Y-chromosomal barcoding of individual sire lines and paved the way for forensic applications. Our robust MSY phylogeny based on biallelic markers will serve as backbone for studying the paternal ancestry of horses on a worldwide scale. Moreover, the incorporation of ancient DNA data should further elucidate the origin of extant lineages in the near future.

## Supplementary information

Supplementary information File

Supplementary Table S1

Supplementary Table S2

Supplementary Table S3

Supplementary Table S4

Supplementary Table S5

Supplementary Table S6

Supplementary Table S7

Supplementary Table S8

Supplementary Table S9

Supplementary Table S10

## Data Availability

The LipY764 contigs can be downloaded from NCBI PRJNA428358 (SUB3434928). Mapped NGS reads of all samples in this paper have been submitted to SRA archive PRJNA430351 (SUB3548921). The newly identified variants, their coordinates and flanking sequences can be found in Supplementary Table S8. Variants being polymorphic in domestic horses and their position detected on eMSYv3 were submitted to ENA (ID will be included in Supplementary Table S8 as soon as received).
