# Supplementary material for: The horse Y chromosome as an informative marker for tracing sire lines
Source: Sci Rep. 2019 Apr 15;9:6095. doi: 10.1038/s41598-019-42640-w (PMC6465346; doi:10.1038/s41598-019-42640-w)
Supplement: Supplementary file 1 — Supplementary information File [file 41598_2019_42640_MOESM1_ESM.pdf]

Supplementary Information File

Manuscript Title:
The horse Y chromosome as an informative marker for tracing sire lines

Authors

Sabine Felkel, Claus Vogl, Doris Rigler, Viktoria Dobretsberger, Bhanu P. Chowdhary, Ottmar Distl, Ruedi Fries, Vidhya Jagannathan, Jan E. Janečka, Tosso Leeb, Gabriella Lindgren, Molly McCue, Julia Metzger, Markus Neuditschko, Thomas Rattei, Terje Raudsepp, Stefan Rieder, Carl-Johan Rubin, Robert Schaefer, Christian Schlötterer, Georg Thaller, Jens Tetens, Brandon Velie, Gottfried Brem and Barbara Wallner

The Supplementary Information File contains

1. Legends to Supplementary Tables
2. Seven Supplementary Figures
3. A detailed description of the methods including codes

1. Legends to Supplementary Tables

Supplementary Table S1: Sample Information

The table provides information on samples used in this study.

Sample ID; breed/taxon; sex; information if sample used for the contig classification (yes/no); mapped file (ID_LipY764_mapped_pp_rmdup_MQ20.bam); SRA biosample ID; data info (single end-SE/paired end-PE); raw data source; SRA whole-genome release (yes/no); number of nucleotides in whole-genome fastq after quality trimming; scY mean coverage; estimated genome coverage; information if the sample was already used in Felkel et al.[1] (yes/no); HT inferred in this study; HT determined in Felkel et al.[1] and Wallner et al.[2].

Supplementary Table S2: Baits used for Y read enrichment

The table lists all Y-chromosomal capture bait sequences. Sources are followed by gene names or sequence IDs in the first column. Columns B and C inform about accessions and species. For each source the total number of sequences and the total Mbp is given in Column D and E.

Supplementary Table S3: scY and mcY windows

Coordinates of scY and mcY windows in the LipY764 assembly.

Contigname; start; end; class and window length (.bed format).

Supplementary Table S4: BLAST nonrepMSY

Results from a BLAST screen of LipY764 and nonrepMSY from Wallner et al.[2]. nonrepMSY contigs were given as ’query’ (Column A), LipY764 as ‘reference’ (Column B). Columns C-K show BLAST results (length of identical sequence tract, the % and the number of identical bases, the start and end of the hit on the query as well as the reference sequence, the e-value and the bitscore of the hit).

Supplementary Table S5: LipY764 contig information

This table contains information for each LipY764 contig.

Columns (A-Q):

Contigname; contig length; length total scY; length total mcY; length total nonMSY; number of scY regions; remarks; number of variants detected on contig; number of SNVs used for dating detected on contig; percentage of contig scY; percentage of contig mcY; percentage of contig nonMSY; number of blast hits unfiltered; number of blast hits when filtering pident > 95 and sident > 300; percentage of contig identical to eMSYv3; eMSYv3 homology category inferred from BLAST results (single hit on eMSYv3, multi-copy on eMSYv3, splitted on eMSYv3, no homology on eMSYv3); total number of bases covered on eMSYv3 by the respective contig.

Columns (R-ES):

BLAST results LipY764 versus eMSYv3 (the percentage and the number of identical bases, the start and end of the hit on the query as well as the reference sequence) of hits with pident > 95 and sident > 300 (number shown in column N).

Supplementary Table S6: Gap distribution

Regions on eMSYv3 not present in LipY764.

Consecutive gaps; gap position start on eMSYv3; gap position end on eMSYv3; length of the gap; sequence category on eMSYv3 as classified in Janečka et al.[3].

Supplementary Table S7: MSY genes on LipY764

Results from a BLAST screen of LipY764 and eMSYv3 transcripts.

eMSYv3 gene ID; transcript ID; start on eMSYv3; end on eMSYv3; length on eMSYv3; orientation on eMSYv3; gene classification according to Janečka et al.[3]; topology according to Janečka et al.[3]; gene conversion predicted in Janečka et al.[3]; proportion of genic region covered with LipY764 found via BLAST; number of LipY764 contigs; ID of LipY764 contigs.

Supplementary Table S8: MSY variant information

The table lists details on all variants used in this study and previously published[1,2].

Variant ID (ID); first described in (study); position on reference (POS); reference allele (REF); alternative allelee (ALT); information if and why the variant was used for dating or not (variant used for dating); major group with the derived allele; haplogroup having the derived allele; samples with positive ALT calls; contig position determined on eMSYv3 using BLAST (if yes: homology category according to Supplementary Table S5); coordinates on eMSYv3; orientation of contig on eMSYv3; information if independent lab validation of the variant was performed (if yes then the technology is given); alternative ID of the variant in other studies (if yes then the study is given); region and gene ID for variants in coding regions; dbSNP/ENA ID; flanking sequence; columns R-FA: allelic state in the donkey (DON) and 139 male horses.

Supplementary Table S9: fBVB microsatellite typing

For 109 analysed samples the table lists: breed; paternal line; haplogroup; allele sizes determined for fBVB.

Supplementary Table S10: Generation time

The table lists 1,771 father-son pairs and their generation interval.

Name of the son; year of birth son; name of the father; year of birth father; generation interval; the haplogroup affiliation of the father-son pair inferred by genotyping a living male descendant; the breed of the present genotyped horse.

2. Supplementary Figures

Supplementary Figure S1

Mean sequencing depth in scY windows. For all 139 males and the donkey the single-copy Y coverage in descending order is shown. The coverage threshold for samples used for dating is 6X and indicated by the crossed lines.

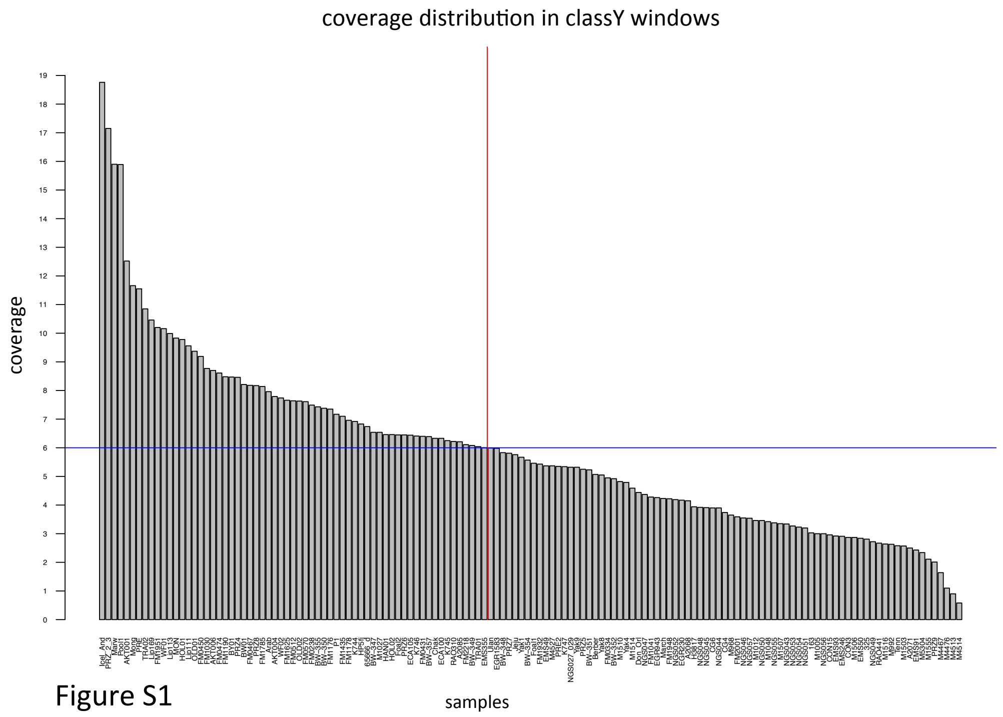

Figure S1

Supplementary Figure S2

Assembly classification. For each contig the Y-specific content is plotted against the contig length. Only contigs larger than 300 bp in length and a Y-specific content > than 45 % were retained in LipY764 (upper right panel).

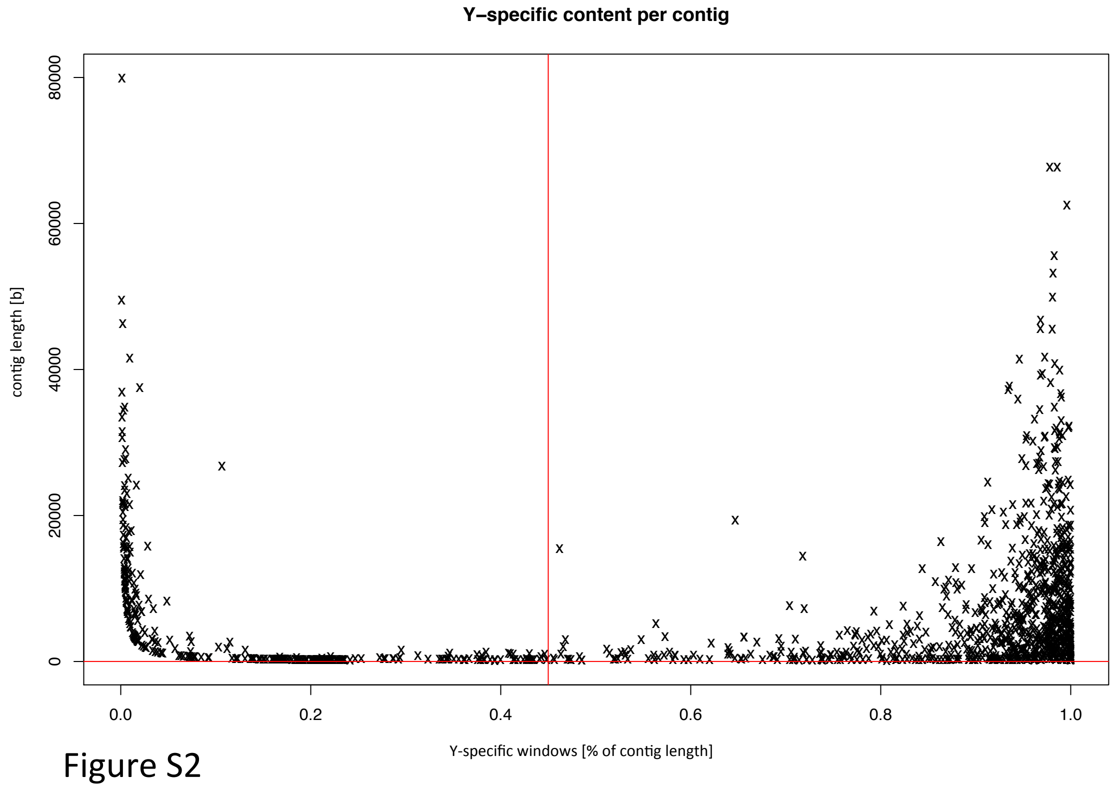

Figure S2

Supplementary Figure S3

LipY764 variant calls after filtering – example when generating a combined vcf for all 139 samples using CombineGVCFs

The first bar shows the number of total calls on LipY764. The number of variants is almost halved when filtering for variants in scY regions only. A large fraction was excluded when the filter for reference errors, phased calls and calls with multiple alternatives was applied. The fourth bar shows the number of variants left when excluding variants with a genotype quality of less than ten and variants that were not covered by more than two reads in at least one individual. The last filtering step involves the exclusion of variants with no or heterozygous calls in more than 10% of the samples. 88 out of the 1,784 final calls were still heterozygous in one are a few samples. When checking these variants manually in IGV, we could validate most of them as homozygous variants being heterozygous in some samples due to mismappings. Note that the resulting number of called variants based on a merged vcf for all 139 samples (as shown here) is 403 less than the final 2,187 variants found on LipY764. As explained in the main text, CombineGVCFs struggles to detect the full diversity for haplotypes underrepresented in the dataset. Hence, to create the definite variant list (Supplementary Table S8) we generated several more vcf's of closely clustering samples (for example only the Przewalski's horses or the deep-splitting Asian and European samples or crown group individuals) and merged the results. The values given here therefore serve as an example of filtering performance when applying the filtering strategy on a vcf containing all samples.

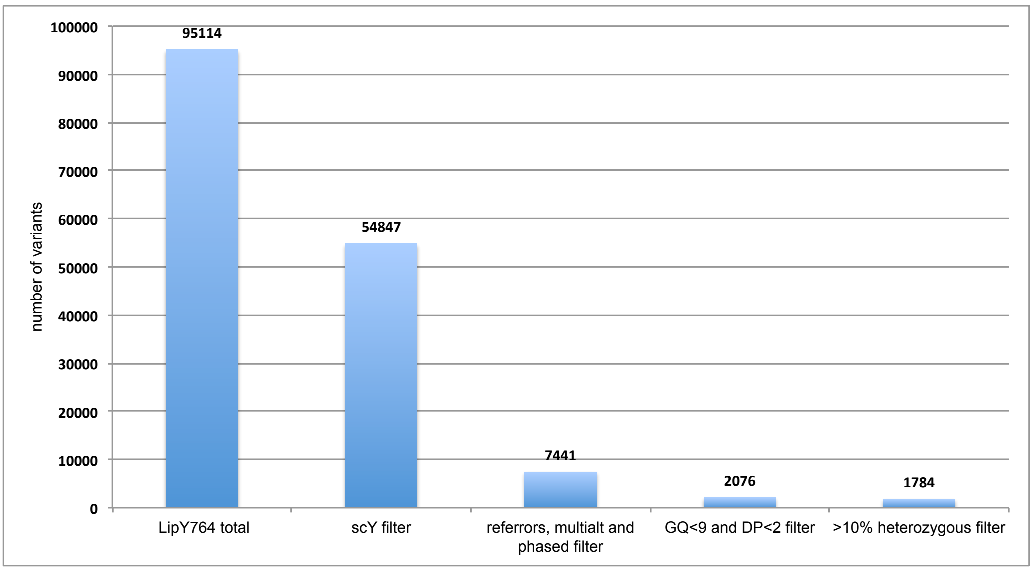

Figure S3

Supplementary Figure S4

MSY haplotype network with variants. A network of all 139 samples with haplotype determining variants shown on branches (in red). Details for all variants can be found in Supplementary Table S8. The circle sizes correspond to the number of samples with that certain haplotype. The crown group is highlighted. Colors correspond to those in Fig. 2. IDs for samples clustering into the respective haplotype are given in Supplementary Table S1.

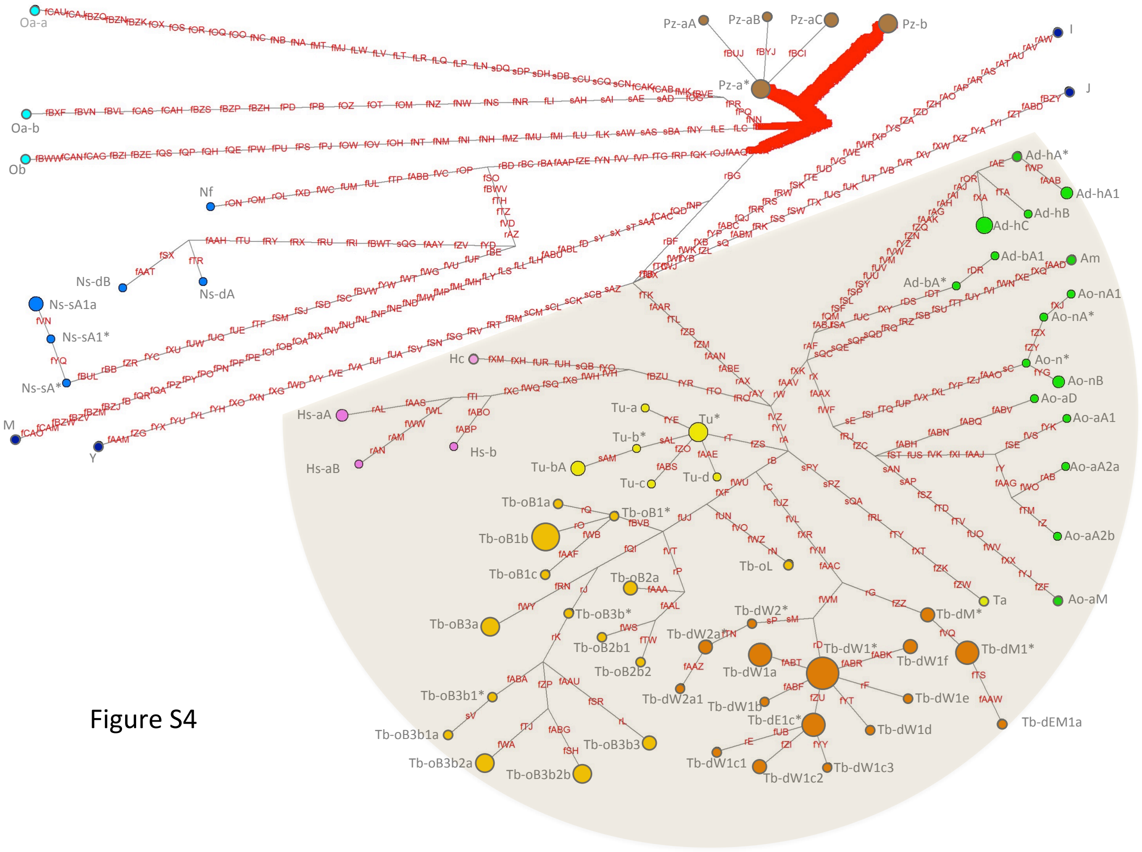

Figure S4

Supplementary Figure S5

A RAxML tree showing the horse MSY phylogeny of 139 males based on the scY variants given in Supplementary Table S8. The tree is rooted with the donkey and bootstrap values are shown. The color-code corresponds to that used in the tree inferred with maximum parsimony shown in Fig. 2.

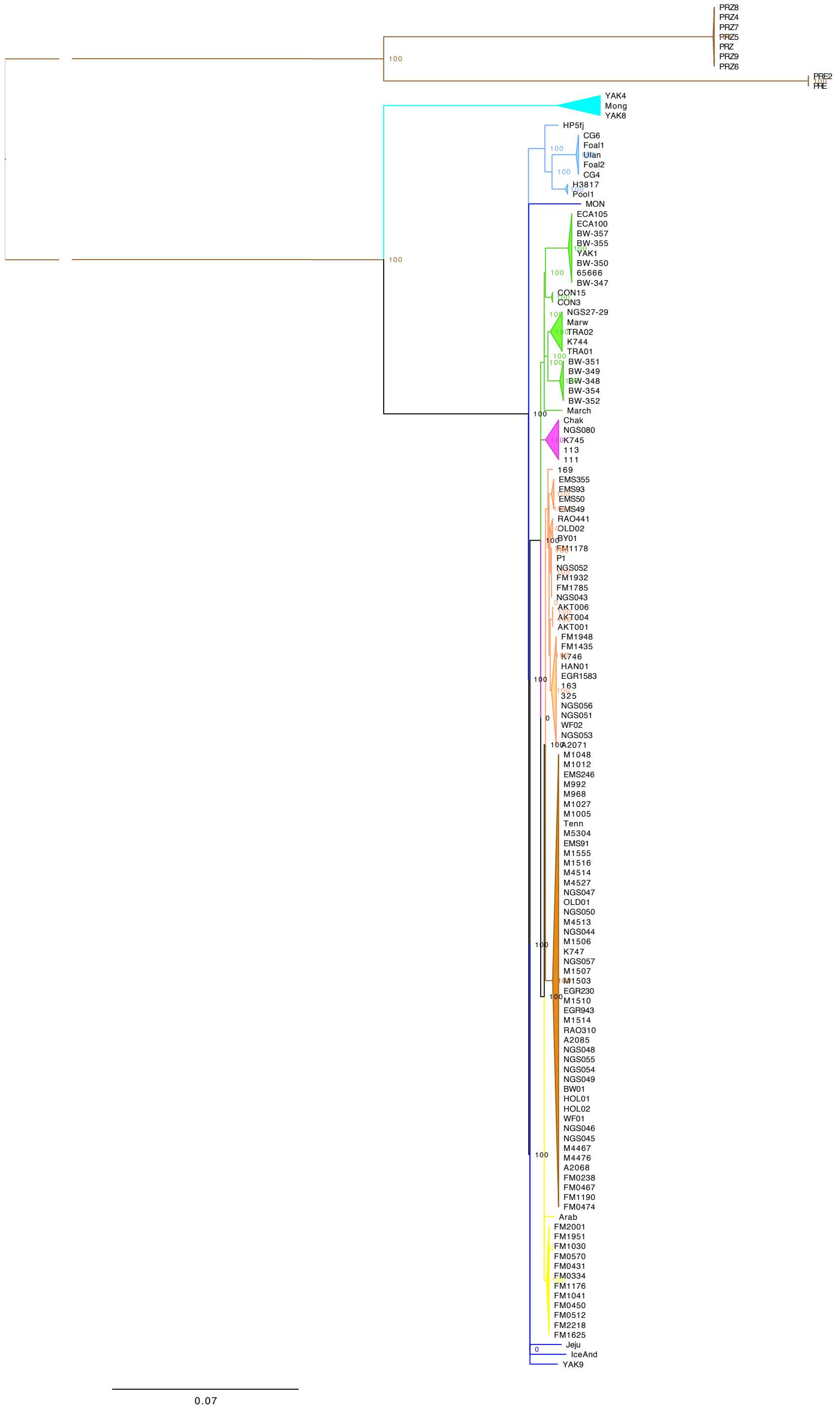

Figure S5

Supplementary Figure S6

Spatial distribution of SNVs used for dating on eMSYv3. For 1,333 of the 1,856 variants used for dating the coordinates on eMSYv3 could be unambiguously determined (dots, coordinates in Supplementary Table S8). Variants on branch Ad are given in blue, on branch M in green. The distance to the closest neighbour was 1,699 bp for fUV and fUU on branch Ad, and 2,616 bp for fOB and fOA on branch M. Since they are on separate LipY764 contigs we could not infer the distance to the closest neighbour for some Ad-h (n=9) and M (n=7) specific variants not present on eMSYv3. For the two pairs on the same LipY764 contig (fOI and fABL on LipY764_contig133; fML and fMP on LipY764_contig50) the distance was 13,636 bp and 19,024 bp, respectively.

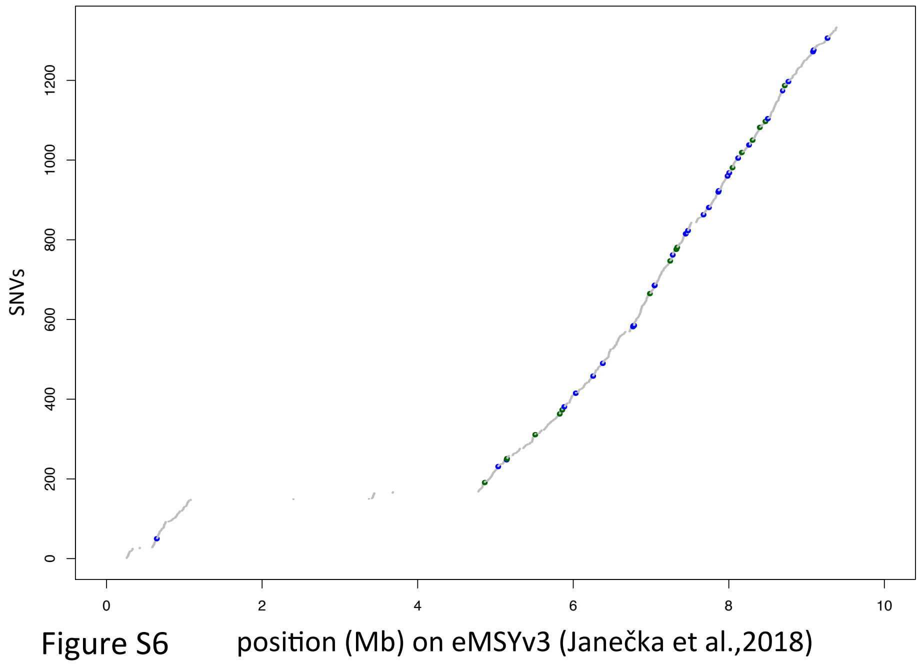

Figure S6

Supplementary Figure S7

Phylogenetic tree resulting from dating with BEAST. This tree is based on 1,856 SNVs (see “variant used for dating” in Supplementary Table S8) found in the 63 samples with a mean scY coverage of at least six (see “scY mean coverage” in Supplementary Table S1). The X axis shows node ages as million years ago (mya). Node bars correspond to the 95% highest posterior density intervals for estimated node dates. The topology corresponds to that inferred with MP and ML methods (Fig. 2 and Supplementary Fig. S5). We named the four most important nodes cab_prz (split of Przewalski’s and present domestic horses), dom_all (all modern domestic horses), dom_west (all modern domestic horses except autochthonous Asian and Northern European lines) and the crown in concordance to Fig. 4.

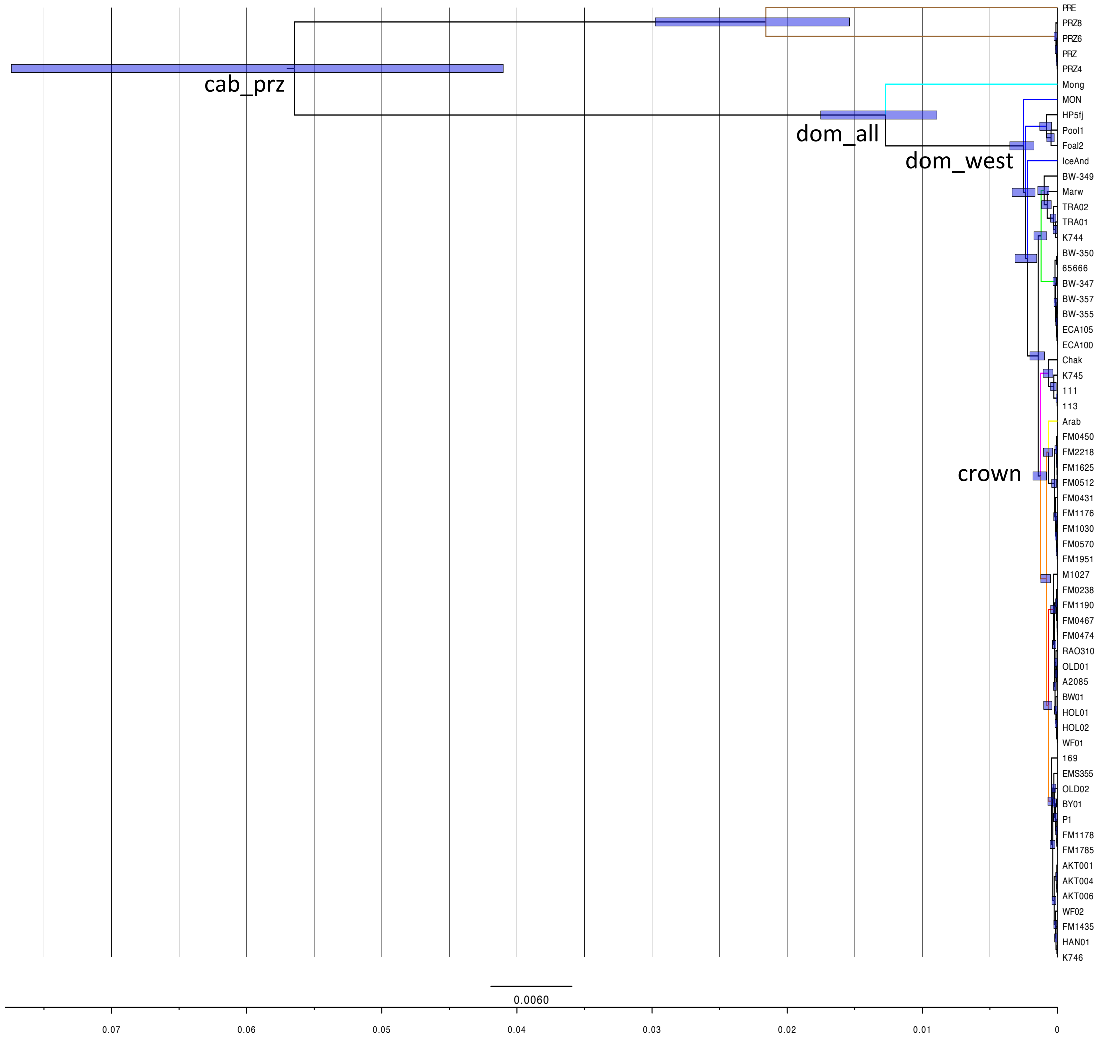

Figure S7

3. Detailed description of methods

Samples and raw data processing

Removing of adaptor sequences and quality-based trimming with ReadTools v0.2.1

```
java -jar ReadTools.jar TrimFastq --input1 INFILE1 --input2 INFILE2 –output OUTFILE
```

Generation of the Y chromosome *de novo* assembly

Y read enrichment

We first extracted putative Y contigs from the whole genome assembly of a Mongolian horse[4] obtained via BLAST v2.4.0+ to the nonrepMSY from Wallner et al.[2].

```
blastn –query NONREPMSY -db MONGOLIANHORSE -outfmt "6 qseqid sseqid length
    pident nident qstart qend sstart send evalue bitscore" -perc_identity 95 -out
    OUTFILE
```

Python v3.5.1 was then used to extract BLAST hits with a minimum length of 150 bp and a minimum identity of 95% to the nonrepMSY.

These contigs, together with other published horse Y-chromosomal sequences – the 1.6 Mbp nonrepMSY[2], six BAC-clones[5], horse Y-chromosomal gene fragments[6] and equine Y-chromosomal and XY-homologous GenBank entries – were merged into a file with multiple FASTA-formatted sequences (bait sequence details listed in Supplementary Table S2). This file was used as reference (bait) to extract Y-specific reads by mapping each Lipizzan male with bwa aln v0.7.15. We removed duplicates and extracted only mapped reads with a minimum mapping quality of 20 and converted them to fasta format with samtools v1.4.

```
bwa aln -t 8 -n 0.02 -l 200 baits.fa INFILE1trim.fq > left.sai
bwa aln -t 8 -n 0.02 -l 200 baits.fa INFILE2trim.fq > right.sai
bwa sampe -r baits.fa left.sai right.sai left_trim.fq right_trim.fq > sample.sam
samtools view -h –b -S -F 4 sample.sam > sample_mapped.bam
samtools sort sample_mapped.bam sample_mapped_sort
samtools rmdup -S sample_mapped_sort.bam sample_mapped_sort_rmdup.bam
samtools view -h -b -F 4 -q 20 sample_mapped_sort_rmdup.bam >
    sample_mapped_sort_rmdup_MQ20.bam
samtools bam2fq sample_mapped_sort_rmdup_MQ20.bam | seqtk seq -A >
    sample_mapped_sort_rmdup_MQ20.fa
```

Python v3.5.1 was used to separate mapped read-pairs into left and right read files.

Assembly

A *de novo* assembly was generated with SPAdes v3.11.1.

```
spades.py --only-assembler --pe1-1 111_left_enriched.fa --pe1-2 111_right_enriched.fa --pe2-1
        113_left_enriched.fa --pe2-2 113_right_enriched.fa --pe3-1 169_left_enriched.fa
        --pe3-2 169_right_enriched.fa -o Lip_Y_raw_Assembly.fa
```

To correct for assembly errors, trimmed whole-genome sequence reads from the three Lipizzans were remapped to the raw assembly using the settings described above. To finally run REAPR v1.0.18 the three files were merged.

```
samtools merge all_Lip_to_Lip_Y_Assembly.bam 111_mapped_sort_rmdup_MQ20.bam
        113__mapped_sort_rmdup_MQ20.bam 169_mapped_sort_rmdup_MQ20.bam
reapr pipeline Lip_Y_raw_Assembly.fa all_Lip_to_Lip_Y_Assembly.bam
        Lip_Y_raw_Assembly_reapr.fa
```

Classification of Y-specific single-copy and multi-copy regions

Mapping

We mapped ten males and five females to the raw assembly and the equine X chromosome with bwa aln v0.7.15, removed duplicates and filtered for mapped reads using samtools v1.4.

```
bwa aln -t 8 -n 0.02 -l 200 Lip_Y+EquCab2_X.fa left_trim.fq > left.sai
bwa aln -t 8 -n 0.02 -l 200 Lip_Y+EquCab2_X.fa right_trim.fq > right.sai
bwa sampe -r Lip_Y+EquCab2_X.fa left.sai right.sai left_trim.fq right_trim.fq >
        sample.sam
samtools view -h –b -S -F 4 sample.sam > sample_mapped.bam
samtools sort sample_mapped.bam sample_mapped_sort
samtools rmdup -S sample_mapped_sort.bam sample_mapped_sort_rmdup.bam
```

Windows

The Y assembly and selected regions of the PAR of the X chromosome (PAR-coordinates were chosen according to Raudsepp and Chowdhary[7] and are given in the table below) were divided into non-overlapping windows of 50 bp length.

PAR gene ID	position on X of Equcab2
PRKX	NC_009175.2:936,109-997,098
MXRA5	NC_009175.2:731,215-761,341
ARSF	NC_009175.2:546,989-573,291
ARSH	NC_009175.2:488,208-517,945
ARSE	NC_009175.2:458,514-484,037
ARSD	NC_009175.2:433,832-453,832
GYG2	NC_009175.2:363,419-399,191
XG	NC_009175.2:312,305-345,217
ZBED1	NC_009175.2:162,769-166,762
ASMT	NC_009175.2:20,985-31,853
DHRSX	NC_009175.2:83,527-98,175

CRLF2	NC_009175.2:103-3,032
PPP2R3B	NC_009175.2:68,000-94,000
GTPBP6	NC_009175.2:41,000-57,000
PLCXD1	NC_009175.2:29,000-39,000

Unix commands and bedtools v2.25.0 were used as given below.

```
cat Lip_Y+EquCab2_X.fa | awk '$0 ~ ">" {print c; c=0;printf substr($0,2,100) "\t"; } $0 !~ ">"
        {c+=length($0);} END { print c; }' > Lip_Y+EquCab2_X_contiglengths.txt
bedtools makewindows -g Lip_Y+EquCab2_X_contiglengths.txt -w 50 >
        Lip_Y+EquCab2_PARwin50.bed
```

Mapping coverage normalisation

Each individual's diploid coverage was inferred as its mean coverage in PAR windows using bedtools v2.25.0. Python v3.5.1 was used to calculate each window's mean coverage. A table with the mean coverages per LipY764 window (row) and individual (column) was provided in the final classification R script (vector "malfem").

```
bedtools coverage -d -a Lip_Y+EquCab2_PARwin50.bed -b sample.bam >
        Lip_Y+EquCab2_PARwin50_cov_per_Parwin50_sample.txt
```

For each horse, the mode of the distribution of the PAR window mean coverages *c* was calculated in R v3.2.3 and used in the final classification R script (vector "mal_par" for males and "fem_par" for females) to normalise the Y assembly mean window coverages such that a relative coverage of one corresponds to a diploid state.

```
#R script to calculate the mode:
contigs=read.table(„sample_meancov.txt", sep = "\t")
        estimate_mode <- function(x) {
          d <- density(x)
          d$x[which.max(d$y)]
        }
        x <- contigs$V4[contigs$V4>0]
        estimate_mode(x)
```

Calculation of female background coverage in confirmed Y regions

The background coverage *b* of Y regions was inferred as the female's mean coverage in windows confirmed to be single-copy Y*.* To predict assured single-copy MSY regions, confirmed Y-specific contigs from Wallner et al.[2] were mapped to the REAPR-corrected Y assembly using bwa mem v0.7.15 (default settings). samtools v1.4 and Unix were used to extract coordinates of the homologous regions.

```
samtools depth nonrepMSY_to_Lip_Y_Assembly_mapped_sort.bam |
```

```
awk '
BEGIN{firsttime=1;}
{
if (pchr!=$1) { if (firsttime==1) { firsttime = 0;} else { printf("%s %d-%d\n",pchr,s,ploc);}s=$2}
else { if ($2!=(ploc+1)){if (ploc!=0){printf("%s %d-%d\n",$1,s,ploc);}s=$2} }
ploc=$2; pchr=$1
}
END{ printf("%s %d-%d\n",pchr,s,ploc);}
' > nonrepMSY_to_Lip_Y_Assembly_covered_regions.bed
```

The mode of the distribution of the window mean coverages per female in these presumably Y-specific regions was calculated (using bedtools v2.25.0 and R v3.2.3) and considered as the female background coverage *b*. These values were provided in the final classification R script (vector "constants").

```
bedtools makewindows -b nonrepMSY_to_Lip_Y_Assembly_covered_regions.bed -w 50 >
    nonrepMSY_to_Lip_Y_Assembly_covered_regions_win50.bed
bedtools coverage -d -a nonrepMSY_to_Lip_Y_Assembly_covered_regions_win50.bed -b
    sample_mapped_sort_rmdup.bam >
    nonrepMSY_to_Lip_Y_Assembly_covered_regions_win50_sample.bed
```

Probabilistic model - derivation of the formula

We first obtained the mean coverage of each male and female horse among all windows that could a priori be assigned to the PAR, which we represent with the symbols c_i for the ith male horse, with $1 \le i \le I$, and c_j for the jth female horse, with $1 \le j \le J$. Next, we obtained the mean background coverage of female horses among all windows that could a priori be assigned to single-copy Y-specific regions, which we represent with b_j for the jth female horse.

Now we assigned the windows of the REAPR-corrected assembly to either MSY or nonMSY according to a likelihood ratio criterion. Assuming that the kth window is within a nonMSY region, and assuming that the coverages y_i and y_j for the male and female horses, respectively, are Poisson distributed, we obtain the following likelihood given the window-specific coverage μ_k:

$$\Pr(y_{i,k}, y_{j,k} \,|\, \mu_k, c) = \prod_{i=1}^{I} \frac{(\mu_k c_i)^{y_{i,k}}\, e^{-\mu_k c_i}}{y_{i,k}!} \prod_{j=1}^{J} \frac{(\mu_k c_j)^{y_{j,k}}\, e^{-\mu_k c_j}}{y_{j,k}!}\,. \qquad (1)$$

The maximum likelihood ratio estimator of the parameter μ_k is:

$$\hat{\mu}_k = \frac{\sum_{i=1}^{I} y_i + \sum_{j=1}^{J} y_j}{\sum_{i=1}^{I} c_i + \sum_{j=1}^{J} c_j} . \tag{2}$$

Assuming that the *k*th window is within the MSY, we obtain the following likelihood:

$$\Pr(y_{i,k}, y_{j,k} \,|\, \nu_k, c, b) = \prod_{i=1}^{I} \frac{(\nu_k c_i/2)^{y_{i,k}}\, e^{-\nu_k c_i/2}}{y_{i,k}!} \prod_{j=1}^{J} \frac{b_k^{y_{j,k}}\, e^{-b_k}}{y_{j,k}!} . \tag{3}$$

The maximum likelihood estimator for the window-specific coverage of the male horses, which we now denote with v_k (to differentiate from the estimator for both male and female horses m_k), is:

$$\hat{\nu}_k = \frac{\sum_{i=1}^{I} y_i}{\sum_{i=1}^{I} c_i/2} . \tag{4}$$

The coverages for the female horses are assumed to correspond to the background frequencies b_j. The likelihood ratio is obtained by substituting the maximum likelihood estimators into the likelihoods:

$$\frac{\Pr(y_{i,k}, y_{j,k} \,|\, \hat{\nu}_k, b, c)}{\Pr(y_{i,k}, y_{j,k} \,|\, \hat{\mu}_k, c)} = \frac{\prod_{i=1}^{I} (\hat{\nu}_k c_i/2)^{y_{i,k}}\, e^{-\hat{\nu}_k c_i/2} \prod_{j=1}^{J} b_k^{y_{j,k}}\, e^{-b_k}}{\prod_{i=1}^{I} (\hat{\mu}_k c_i)^{y_{i,k}}\, e^{-\hat{\mu}_k c_i} \prod_{j=1}^{J} (\hat{\mu}_k c_j)^{y_{j,k}}\, e^{-\hat{\mu}_k c_j}} . \tag{5}$$

Probabilistic model - calculation

The LipY764 mean coverages per window and sample (table “malfem”) and the mean PAR window coverages for males (vector “mal_par”) and females (vector “fem_par”), to normalise the former, are provided as input for the R script. Also, the female background coverages (vector “constants”) are provided. Calculations have been performed with R v3.2.3.

```
# classification R script:
    malfem=read.table("males+females_win50_meancov.txt")
    fem_par <- c(provide meanPARwindowcoverages:female1,female2,(…),femaleX)
    mal_par <- c(provide meanPARwindowcoverages:male1,male2,(…),maleX)
    constants <- c(provide meannonrepMSYwindowcoverages:female1,female2,(…),femaleX)
```

```
nwindows=length(malfem[,1])
start=1
estimated_1_m=estimated_mf5=estimated_mf5_m1f2=rep(-1,nwindows)
total_likeli1=total_likeli2=total_likeli3=rep(0.5,nwindows)
for(i in start:nwindows){
#define the columns with male data → 4 to 13 in our case
  mal=as.numeric(malfem[i,4:13])
#define the columns with female data → 14 to 18 in our case
  fem=as.numeric(malfem[i,14:18])
  malfem=c(mal,fem)
  malfem_par=c(mal_par,fem_par)
  estimated_1_m[i]=m1=sum(mal)/sum(mal_par)
  estimated_mf5[i]=m2=sum(malfem)/sum(malfem_par)
  estimated_mf5_m1f2[i]=m3=sum(malfem)/(sum(mal_par)+sum(fem_par))
  if((m1>0) & (m2>0)){
#here you calculate the window's likelihood for being MSY and nonMSY:
    total_likeli1[i]=sum(mal*log(m1*mal_par)-m1*mal_par)+sum(fem*log(constants5)-constants5)
    total_likeli2[i]=sum(malfem*log(m2*malfem_par)-m2*malfem_par)
  }
}
#now extract windows that have higher likelihood to be MSY:
prob1=1/(exp(total_likeli2 - total_likeli1)+1)
idx=prob1>0.5
hist(estimated_1_m[idx],xlim=c(0,25),ylim=c(0,30000),nclass=250,main=paste("histogram of Y-
       specific windows"),xlab="relative copynumber",ylab="number of windows")

#threshold 1 was chosen based on hist above, can be adjusted
idx1=prob1>0.5 & estimated_1_m<1
idx2=prob1>0.5 & estimated_1_m >=1
#save single-copy and multi-copy MSY windows as separate lists
window_id=data.frame(malfem[,1][idx1],malfem[,2][idx1],malfem[,3][idx1])
write.table(window_id,file="Lip_Y_Assembly_scY_windows.txt")
window_id=data.frame(malfem[,1][idx2],malfem[,2][idx2],malfem[,3][idx2])
write.table(window_id,file="Lip_Y_Assembly_mcY_windows.txt")
```

A relative coverage cut-off value less than one was selected to distinguish scY from mcY windows (see Supplementary Fig. S2).

LipY764 gene content and homologies to nonrepMSY and eMSYv3

BLAST v.2.4.0+ was used to align LipY764 to

- nonrepMSY from Wallner et al.[2] (query nonrepMSY, reference LipY764)
- the eMSYv3 from Janečka et al.[3] (query LipY764, reference eMSYv3)

We set the thresholds for a perfect match to nident > 300 and pident > 95%.

Variant calling and generation of the horse Y phylogeny

Adaptor free and trimmed data (Supplementary Table S1) were mapped to the raw assembly after classification using bwa aln v0.7.15. Unmapped reads, PCR duplicates and low quality mappings were filtered with samtools v1.4. Variant calling was performed using GenomeAnalysisTK v3.7 HaplotypeCaller and CombineGVCFs.

```
#in case of SE data run bwa aln only once and use bwa samse instead of sampe
bwa aln -t 8 -n 0.02 -l 200 Lip_Y_Assembly_reapr.fa left_trim.fq > left.sai
bwa aln -t 8 -n 0.02 -l 200 Lip_Y_Assembly_reapr.fa right_trim.fq > right.sai
bwa sampe -r Lip_Y_Assembly_reapr.fa left.sai right.sai left_trim.fq right_trim.fq > sample.sam
samtools view -h –b -S -F 4 sample.sam > sample_mapped.bam
samtools sort sample_mapped.bam sample_mapped_sort
samtools rmdup -S sample_mapped_sort.bam sample_mapped_sort_rmdup.bam
samtools view -h -b -F 4 -q 20 sample_mapped_sort_rmdup.bam >
        sample_mapped_sort_rmdup_MQ20.bam
java -jar GenomeAnalysisTK.jar -T HaplotypeCaller –I sample_mapped_sort_rmdup_MQ20.bam
        -R Lip_Y_Assembly_reapr.fa > sample.g.vcf
java -jar GenomeAnalysisTK.jar -T CombineGVCFs -V sample1.g.vcf -V sample2.g.vcf
        –V sampleX.g.vcf -o cohort.g.vcf
```

Pedigree reconstruction

Paternal genealogies were inferred using pedigree information provided by breeding associations or by reconciling results from multiple databases. All databases were last accessed in May 2018.

Databases used:

www.bloodlines.net
www.pedigreequery.com
www.galopp-sieger.de
www.shagya-database.ch
www.stormhestar.de
www.pedigreequery.com
www.sporthorse-data.com
www.allbreedpedigree.com
www.zsaa.org
www.haflingerhorse.com
www.bazakoni.pl
www.pferdezucht-austria.at
www.sukuposti.net
www.stavropol-teke.ru
www.hucul-achhk.cz
www.aschk.cz

www.mlosz.hu
www.rimondo.com

MSY HG determination

For 333 male horses, with pedigrees available, the MSY HG was determined by genotyping key variants (see below and Supplementary Fig. S4). All samples were derived from breeding associations and private horse owners. Genomic DNA was isolated from hair roots using nexttecTM®. For genotyping, competitive allele-specific PCR SNV genotyping assays (KASP™, lgcgroup.com) were used. KASP™ screening was performed as described (lgcgroup.com) on a CFX96 Touch™ Real-Time PCR Detection System. Female samples were included to check MSY specificity of the KASP-assays.

haplogroup	derived allele	ancestral allele
Ad:	rW, rAF	rA, fRO
Ao:	rW, rX	rA, fRO
Tb-d:	rA, rC, rG*, sM*, rD*	rW, fRO
T non Tb-d:	rA, rB*, rT*, sPY*, rP*, rK*	rW, fRO, rC
H:	fRO	rA, rW
I:	rBF, rBG, rAW	rAX, rA, rW, fRO
N:	rBA	rBF, rBG

* preclusive alleles

Test for equality of numbers of mutations on branches

Equality of numbers of mutations on pairs of branches originating from the same node was checked using a chi-square test; p-values were determined by 10,000 simulations in R v3.2.3.

- For example, starting from the basal crown node we consider the 26 mutations on the longest branch leading to an observed Ad haplotype vs. the five on the shortest branch leading to an observed Tu haplotype (see Fig. 4):

```
chisq.test(cbind(26,5),p=c(0.5,0.5),simulate.p.value=TRUE,B=10000)
```

Additional tests were performed considering

- all branches evolving from the basal crown node:

```
chisq.test(cbind(5,7,8,9,10,11,12,13,14,15,17,24,26),simulate.p.value=TRUE,B=10000)
```

- all branches evolving from the basal dom_west node:

```
chisq.test(cbind(14,16,17,18,19,20,21,22,23,24,25,26,32,33,35,40),simulate.p.value=TRUE,B=100
00)
```

- all branches evolving from the basal dom_all node:

```
chisq.test(cbind(124,126,127,128,129,130,131,132,133,134,136,142,143,145,146,150),simulate.p.v
alue=TRUE,B=10000)
```

- all branches evolving from the basal dom_prz node:

```
chisq.test(cbind(546,548,549,550,551,552,553,554,555,556,557,558,564,565,567,572,599,568,615
),simulate.p.value=TRUE,B=10000)
```

To account for the different generation intervals, we ran another chi-square test accounting for these differences with simulated p-value based on 10,000 replicates.

- For the two branches mentioned above (Ad-h=26 mut and the shortest T branch=5 mut), and the respective mean generation intervals of 8.935294 for Ad carriers and 12.225434 for T carriers (Fig. 4b), the test becomes:

```
expected=c(1/8.935294,1/12.225434)/sum(c(1/8.935294,1/12.225434))
chisq.test(cbind(26,5),p=expected,simulate.p.value=TRUE,B=10000)
```

Mutation rate estimate

For mutation rate calculation we assume:

- mean generation interval in Tb-d father-son pairs = 11.36 years
- mean number of mutations in Tb-d after Darley Arabian (320 years/28.17 generations ago)= 2.78
- total length scY windows = 5,834,017

Accordingly we infer:

- mutations per meiosis in region under investigation: 2.78/28.17 = 0.0986865
- one mutation every = 10.13 meiosis/generations (1/0.0986865*)*
- mutation rate per site per meiosis: 0.0986865/5834017 = 1.6916×10^{-8}

Supplementary Information References

1. Felkel, S. *et al.* Asian horses deepen the MSY phylogeny. *Anim. Genet.* **49,** 90–93 (2018).
2. Wallner, B. *et al.* Y Chromosome Uncovers the Recent Oriental Origin of Modern Stallions. *Curr Biol.* **Jul 10;27,** 2029–2035 (2017).
3. Janečka, J. E. *et al.* Horse Y chromosome assembly displays unique evolutionary features, putative stallion fertility genes and horizontal transfer. *Nat. Commun.* **9,** 2945 (2018).
4. Huang, J. *et al.* Analysis of horse genomes provides insight into the diversification and adaptive evolution of karyotype. *Sci. Rep.* **4,** 1–8 (2014).
5. Wallner, B. *et al.* Identification of Genetic Variation on the Horse Y Chromosome and the Tracing of Male Founder Lineages in Modern Breeds. *PLoS One* **8,** (2013).
6. Paria, N. *et al.* A gene catalogue of the euchromatic male-specific region of the horse y chromosome: comparison with human and other mammals. *PLoS One* **6,** e21374 (2011).
7. Raudsepp, T. & Chowdhary, B. P. The horse pseudoautosomal region (PAR): characterization and comparison with the human, chimp and mouse PARs. *Cytogenet Genome Res* **121,** 102–109 (2008).

List of tools used

Tool	Reference/Link
BEAST v.1.8.1	Suchard MA, Lemey P, Baele G, Ayres DL, Drummond AJ & Rambaut A (2018) Bayesian phylogenetic and phylodynamic data integration using BEAST 1.10 Virus Evolution 4, vey016. DOI:10.1093/ve/vey016
bedtools v2.25.0	Quinlan, A. R. & Hall, I. M. BEDTools : a flexible suite of utilities for comparing genomic features. Bioinformatics 26, 841–842 (2010).
BLAST v.2.6.0	https://blast.ncbi.nlm.nih.gov/
bwa v0.7.15-r1140	Li, H. & Durbin, R. Fast and accurate short read alignment with Burrows – Wheeler transform. Bioinformatics 25, 1754–1760 (2009).
CLC Genomics Workbench 7.7.1	https://www.qiagenbioinformatics.com/
Datamonkey	http://classic.datamonkey.org/dataupload.php
FigTree v1.4.2	http://tree.bio.ed.ac.uk/software/figtree/
GenomeAnalysisTK v3.7	McKenna, A. et al. The Genome Analysis Toolkit: a MapReduce framework for analyzing next-generation DNA sequencing data. Genome Res. 20, 1297–303 (2010).
IGV v2.3.68	https://software.broadinstitute.org/software/igv/
java v1.8.0_91	http://www.oracle.com/technetwork/java/index.html
ncbi-blast v2.4.0+	Camacho, C. et al. BLAST+: architecture and applications. BMC Bioinformatics 15, 521 (2009).
Network v4.614	http://www.fluxus-engineering.com/sharenet.htm
PAUP v4.0a	http://paup.phylosolutions.com/
picard-tools v2.3.0	http://broadinstitute.github.io/picard
Python v3.5.1	http://www.python.org
R v3.2.3	R Core Team (2017). R: A language and environment for statistical computing. R Foundation for Statistical Computing, Vienna, Austria. URL http://www.R-project.org/
RAxML v.8.1.13	https://cme.h-its.org/exelixis/web/software/raxml/index.html
ReadTools v.0.2.1.r_716422a3	Gómez-Sánchez, D. & Schlötterer, C. ReadTools: A universal toolkit for handling sequence data from different sequencing platforms. Mol. Ecol. Resour. 18, 676–680 (2018).
REAPR v1.0.18	Hunt, M. et al. REAPR: A universal tool for genome assembly evaluation. Genome Biol. 14, (2013).
samtools v1.4	Li, H. et al. The Sequence Alignment / Map format and SAMtools. Bioinformatics 25, 2078–2079 (2009).
seqtk v1.2-r94	seqtk, Toolkit for processing sequences in FASTA/Q formats. Available from: https://github.com/lh3/seqtk.
SPAdes v3.11.1	Bankevich, A. et al. SPAdes: A New Genome Assembly Algorithm and Its Applications to Single-Cell Sequencing. J. Comput. Biol. 19, 455–477 (2012).
Sratoolkit v2.8.2	https://www.ncbi.nlm.nih.gov/sra/docs/toolkitsoft/
